# USP13 regulates HMGB1 stability and secretion through its deubiquitinase activity

**DOI:** 10.1186/s10020-022-00596-0

**Published:** 2022-12-30

**Authors:** Jaemin Shin, Young Hun Kim, Bin Lee, Jae Ho Chang, Hee Youn Choi, Hoojung Lee, Ki Chan Song, Man Sup Kwak, Ji Eun Choi, Jeon-Soo Shin

**Affiliations:** 1 Department of Microbiology, Yonsei University College of Medicine, 50-1 Yonsei-Ro, Seodaemun-Gu, Seoul, 03722 South Korea; 2 Brain Korea 21 FOUR Project for Medical Science, Yonsei University College of Medicine, Seoul, 03722 South Korea; 3 Institute for Immunology and Immunological Diseases, Yonsei University College of Medicine, Seoul, 03722 South Korea; 4 Department of Pediatrics, Seoul National University College of Medicine, Seoul Metropolitan Government Seoul National University Boramae Medical Center, Boramaero 5 Gil 20, Dongjakgu, Seoul, 07061 South Korea

**Keywords:** HMGB1, Deubiquitination, USP13, Secretion, Spautin-1

## Abstract

**Background:**

High mobility group box 1 (HMGB1) is a damage-associated molecular pattern (DAMP) molecule that plays a central role in innate immunity. HMGB1 acts as a late mediator of inflammation when actively secreted in response to inflammatory stimuli. Several post-translational modifications (PTMs), including acetylation, phosphorylation, and oxidation, are involved in HMGB1 secretion. However, the E3 ligases of HMGB1 and the mechanism by which DUBs regulate HMGB1 deubiquitination are not well known.

**Methods:**

LC–MS/MS, proximity ligation assay, immunoprecipitation were used to identify ubiquitin-specific protease 13 (USP13) as a binding partner of HMGB1 and to investigate ubiquitination of HMGB1. USP13 domain mutant was constructed for domain study and Spautin-1 was treated for inhibition of USP13. Confocal microscopy image showed localization of HMGB1 by USP13 overexpression. The data were analyzed using one-way analysis of variance with Tukey’s honestly significant difference *post-hoc* test for multiple comparisons or a two-tailed Student’s *t*-test.

**Results:**

We identified ubiquitin-specific protease 13 (USP13) as a novel binding partner of HMGB1 and demonstrated that USP13 plays a role in stabilizing HMGB1 from ubiquitin-mediated degradation. USP13 overexpression increased nucleocytoplasmic translocation of HMGB1 and promoted its secretion, which was inhibited by treatment with Spautin-1, a selective inhibitor of USP13.

**Conclusion:**

Taken together, we suggest that USP13 is a novel deubiquitinase of HMGB1 that regulates the stability and secretion of HMGB1.

## Introduction

High mobility group box 1 (HMGB1) is a nuclear protein highly conserved in most eukaryotic cells (Laudet et al. 1993). In the nucleus, HMGB1 regulates gene transcription and DNA repair, and it functions as a DNA chaperone to modulate nucleosome stability (Javaherian et al. 1978; Lange and Vasquez 2009; Stros et al. 2002). In the extracellular space, HMGB1 acts as a damage-associated molecular pattern (DAMP), a late mediator of the pro-inflammatory response (Wang et al. 2001) and also it early secreted in some trauma like burn (Lantos et al. 2010). HMGB1 is actively secreted in response to various stimuli, such as lipopolysaccharide (LPS), interleukin (IL)-1, tumor necrosis factor (TNF)-α, and hydrogen peroxide (H_2_O_2_) (Tang et al. 2007; Wang et al. 1999, 2004; Yang et al. 2004). Following stimuli, post-translational modifications (PTMs), including oxidation, acetylation, phosphorylation, and methylation, are involved in the regulation of HMGB1 secretion (Bonaldi et al. 2003; Hoppe et al. 2006; Ito et al. 2007; Youn and Shin 2006). Recently, it was found that peroxiredoxin-I/II is involved in facilitating HMGB1 oxidation in the nucleus (Kwak et al. 2019) and that secretory autophagy with vesicular trafficking pathways is involved in its secretion (Kim et al. 2021).

Ubiquitination, a PTM, plays a key role in the regulation of protein stability and signal transduction pathways. Ubiquitination is a series of processes that conjugate ubiquitin to the target substrate in a reversible and ATP-dependent manner. This process is achieved by E1 (the ubiquitin-activating enzyme), E2 (ubiquitin-conjugating enzyme), and E3 (ubiquitin ligases) enzymatic cascade (Pickart and Eddins 2004). Deubiquitination is the reversal of ubiquitination (Amerik and Hochstrasser 2004). The human genome encodes approximately 100 deubiquitinases (DUBs), which are opposed to E3 ligases. DUBs are classified into five families based on their sequence homology and mechanism of action: ubiquitin-specific proteases (USPs), ubiquitin C-terminal hydrolases (UCHs), ovarian tumor proteases (OTUs), Machado-Joseph disease proteases, and JAB1/MPN/Mov34 metalloenzymes (JAMMs) (Nijman et al. 2005). Correspondingly, the functions of DUBs can be divided into several categories. First, DUBs process ubiquitin precursors to generate free ubiquitin. Second, DUBs remove K48-linked ubiquitin chains, which are involved in proteasomal degradation, leading to the stabilization of substrate proteins. Lastly, DUBs are also involved in non-degradative ubiquitin signaling by trimming ubiquitin chains while they switch from one type of ubiquitin signal to another. Consequently, all of these functions of DUBs contribute to the regulation of ubiquitin homeostasis to maintain cellular ubiquitin pools (Ye et al. 2009). USPs contain a catalytic core domain, including an N-terminal Cys-box and C-terminal His-box, which consist of the catalytic Cys and His residues, respectively (Ye et al. 2009). Furthermore, USPs have other functional domains flanking or inserting into the core domain, such as the zinc finger (ZnF) domain, ubiquitin-interacting motif (UIM), and ubiquitin-associated (UBA) domains (Zhang et al. 2011). The UBA and UIM domains allow the substrate to bind to enzymes, whereas the ZnF domain might activate the hydrolytic reactions and regulate enzyme activity (Bonnet et al. 2008).

Ubiquitin-specific protease 13 (USP13) belongs to the USP family and shows  sequence similarity up to 80% with USP5. These two USPs also share the same domains, including a ZnF domain, a catalytic core composed of a C-box and H-box, and two UBA insertions (Zhang et al. 2011). USP13 modulates cellular signaling by interacting with multiple proteins. For example, USP13 regulates antiviral responses by deubiquitinating STAT1 and STING (Sun et al. 2017; Yeh et al. 2013) and is related to PTEN and MITF in the modulation of tumorigenesis (Zhang et al. 2013; Zhao et al. 2011). In addition, RAP80-BRCA1 complex is regulated by USP13 in response to DNA damage (Li et al. 2017).

Although several PTMs modulate HMGB1, ubiquitination of HMGB1 is poorly understood. Our previous study revealed that defective HMGB1 glycosylation regulates HMGB1 stability through K48-linked ubiquitination (Kim et al. 2016). However, the E3 ligases of HMGB1 remain unknown. Furthermore, the mechanism by which DUBs regulate HMGB1 deubiquitination is unknown. Here, we report USP13 as a novel protein that interacts with HMGB1. We demonstrated that USP13 deubiquitinates and stabilizes HMGB1. Unexpectedly, we found that USP13 regulated HMGB1 secretion via its deubiquitinase activity. Overall, this study revealed a novel USP13-HMGB1 pathway regulating HMGB1 stability and secretion.

## Material and methods

### Cell culture and transfection

Human embryonic kidney (HEK) 293 T (ATCC® CRL-1573™), RAW 264.7, (ATCC® TIB-71™), and HEK293/hTLR4-MD2-CD14 (InvivoGen, San Diego, CA, USA) cells were cultured in DMEM media supplemented with 10% FBS (Corning Cellgro, Tewksbury, MA, USA), 2 mM L-glutamine, 100 U/mL penicillin, and 100 μg/mL streptomycin at 37 °C with 5% CO_2_. HEK293/hTLR4-MD2-CD14 cells were supplemented with 10 μg/mL blasticidin and 50 μg/mL hygromycin (InvivoGen). Plasmids were transfected into HEK293T cells using lipofectamine 2000 (Invitrogen, Carlsbad, CA, USA) according to the manufacturer’s instructions.

### Plasmid construction

Myc and GFP-HMGB1 in pCMV and pEGFP–N1 vectors, respectively, were used (Kim et al. 2016). pRK5-FLAG-USP13 (Addgene plasmid #61741; http://n2t.net/addgene:61741; RRID: Addgene_61741), Flag-HA-USP10 (Addgene plasmid #22543; http://n2t.net/addgene:22543; RRID: Addgene_22543), HA-Ub (Addgene plasmid #18712; http://n2t.net/addgene:18712; RRID: Addgene_18712), and pRK5-HA-Ub-K48R (Addgene plasmid #17604; http://n2t.net/addgene:17604; RRID: Addgene_17604). Flag-HA-USP10 was subcloned into pFLAG-CMV-TOPO vector (MGmed, Seoul, Korea, MC02204). Flag-USP15 plasmid was kindly provided by Prof. Kyung-Hee Chun (Yonsei University, Seoul, Korea). USP13 deletion mutants of the Flag-USP13-ZnF (amino acid 178–301) and USP13-USP (amino acid 336–863) (YH Zhang et al. 2011) were constructed. The USP13^C345A/M664/739E^ inactive mutant (USP13 Mut) was generated using the QuickChange Site-Directed Mutagenesis Kit (Stratagene, San Diego, CA, USA). To transiently knock down USP13, Human USP13 shRNAs were transiently knocked down in a GIPZ lentiviral vector (Dharmacon Inc., Lafayette, CO, USA; clone No. V2LHS_47077).

### Antibodies and chemicals

Antibodies against HMGB1 (Abcam, Cambridge, UK, ab18256), USP13 (Bethyl, Montgomery, TX, USA, A302-762A), β-actin (Cell Signaling Technology, Danvers, MA, USA, #4967), Ub (Millipore, Billerica, MA, USA, MAB1510), Ub-K48 (#05-1307), Ub-K63 (#05-1308), c-Myc (Invitrogen Technology, #13-2500), Flag (Sigma-Aldrich, St. Louis, MO, USA, F7425), HA (Santa Cruz Biotechnology, Santa Cruz, CA, USA, sc-805), and CRM1 (Santa Cruz Biotechnology, sc-5595) were used. Spautin-1 (Tocris, Bristol, UK, 5197), a USP13 inhibitor, was used at a concentration of 10 μM (Liu et al. 2011). A 10 μM MG132 proteasome inhibitor (Sigma-Aldrich, C2211), 100 μg/mL cycloheximide (CHX) (Sigma-Aldrich, C7698) protein biosynthetic inhibitor, 20 ng/mL leptomycin B (LMB, Sigma Aldrich, L2913) a CRM1 inhibitor, and 80 nM wortmannin (Sigma-Aldrich, W1628) of PI3K inhibitor were used. To induce HMGB1 secretion, 100 ng/mL LPS (*E. coli* O111:B4, Sigma-Aldrich, L4391) and H_2_O_2_ (Merck, Kenilworth, New Jersey, USA, 1.07209.0250) were used.

### Immunoblotting (IB) and immunoprecipitation (IP)

HEK293T cells were co-transfected with the various plasmids and washed with PBS. For immunoblotting, the cells were lysed with 1 × RIPA buffer (GenDEPOT, Barker, TX, USA) containing a protease inhibitor cocktail (GenDEPOT). After sample buffer (100 mM Tris–HCl pH 6.8, 25% glycerol, 5% β-mercaptoethanol, 2% SDS, and 0.1% bromophenol blue) was added to whole cell lysates (WCLs), the mixture was subsequently heated at 94 °C for 5 min. Quantitative proteins were separated by SDS-PAGE and transferred to nitrocellulose membranes (GE Healthcare, Little Chalfont, UK), which were blocked with 5% skim milk in Tris-buffered saline (TBS) with 0.1% Tween-20. The indicated antibodies were incubated, and ECL solution (GenDEPOT) was used for detection. To detect HMGB1 secretion, cell culture supernatants were harvested after treatment, and a methanol and chloroform mixture at a 4:1 ratio was added to supernatants to precipitate proteins, and then immunoblotted. For IP, pre-cleared protein G magnetic beads (Bio-Rad, Hercules, CA, USA) were reacted with the indicated antibodies for 1 h at room temperature (RT), and WCLs were incubated with beads overnight at 4 °C. After the beads were washed four times with PBST buffer (0.001% Tween-20 in PBS), the immunoprecipitates were eluted with the sample buffer and separated by SDS-PAGE.

### Ubiquitination assay

HEK293T cells were co-transfected with Myc-HMGB1, HA-Ub, and Flag-USP13 wild-type (WT) or Flag-USP13 Mutant. After transfection for 24 h, cells were treated with 10 μM MG132 for 18 h. Cells were lysed, and protein G magnetic beads conjugated with anti-Myc Ab were added to the sample. The eluted samples were resolved by SDS-PAGE, and anti-Ub and HA antibodies were incubated overnight at 4 °C for immunoblotting.

### Immunofluorescence assay

To observe the subcellular translocation of HMGB1, GFP-HMGB1, and Flag-USP13 WT or Flag-USP13 Mut were co-transfected into HEK293T cells, which were cultured on cover glasses in a six-well plate. After 24 h, cells were pre-treated with 10 μM Spautin-1 for 2 h and then treated with 50 μM H_2_O_2_ for 4 h. The cells were fixed with 4% paraformaldehyde-PHEM buffer for 30 min at RT and permeabilized with 1% Triton X-100 for 10 min at RT. After washing with cold PBS, the cells were blocked with 1% BSA in PBST buffer (0.02% Tween-20 in PBS) for 30 min at 37 °C, and then incubated with anti-FLAG Ab overnight at 4 °C, followed by incubation with Alexa Fluor 594 goat anti-mouse IgG (Life Technologies, A11005) for 45 min at 37 °C. After mounting with DAPI (Vector Laboratories, Burlingame, CA, USA), a confocal FV1000 microscope (Olympus, Shinjuku, Tokyo, Japan) was used to generate images.

### Proximity ligation assay (PLA)

To confirm the interaction between HMGB1 and USP13, PLA was performed using the Duolink in situ fluorescence kit (Sigma-Aldrich). HEK293T cells were co-transfected with Myc-HMGB1 and Flag-USP13 plasmids for 18 h and treated with 10 μM Spautin-1 for 18 h. After fixation and permeabilization, the cells were blocked with a blocking solution. Cells were incubated with anti-Myc and anti-FLAG antibodies overnight at 4 °C. Next, PLA probes and ligation solutions were reacted. After treatment with the amplification solution, cells were mounted with DAPI. Finally, a confocal microscope was used to measure cell fluorescence.

### Nuclear/cytosolic fractionation

HEK293T cells were lysed using a nuclear/cytosol fractionation kit (Bio Vision, K266-100) according to the manufacturer’s instructions. The nuclear and cytosolic fractions were resolved by SDS-PAGE.

### Liquid chromatography with tandem mass spectrometry (LC-MS/MS) for the analyses of peptides

HEK293T cells were co-transfected with Myc-HMGB1 and HA-Ub plasmids. Cells were lysed with 1 × RIPA buffer and incubated with anti-Myc affinity gel (Biotool, Houston, TX, USA, B23401) overnight at 4 °C. The resin was washed with cold PBS three times and a 2 × sample buffer was added to elute the sample. After boiling at 94 °C for 5 min, the samples were separated by SDS-PAGE, followed by Coomassie Blue staining. The band located at the putative HMGB1 binding partner was extracted for analysis. Nano LC-MS/MS analysis was performed using a nano high performance liquid chromatography system (Agilent, Wilmington, DE, USA). A Nanochip column (Agilent, 150 mm × 0.075 mm) was used for peptide separation. The mobile phase A for LC separation was 0.1% formic acid in deionized water and mobile phase B was 0.1% formic acid in acetonitrile. The chromatographic gradient was designed for a linear increase from 3% B to 40% B in 80 min, 40% B to 60% B in 10 min, 95% B in 10 min, and 3% B in 20 min. The flow rate was maintained at 400 nL/min. Product ion spectra were collected in the information-dependent acquisition mode and analyzed by Agilent 6530 Accurate-Mass Q-TOF using continuous cycles of one full scan TOF MS of 350–1200 m/z (1.0 s) plus three product ion scans from to 100–1700 m/z (1.5 s each). Precursor m/z values were selected starting with the most intense ion using a selection quadrupole resolution of 4 Da. The rolling collision energy feature was used to determine the collision energy based on the precursor value and charge state. The dynamic exclusion time for the precursor ion m/z values was 30 s.

### Database searching

The Mascot algorithm (Matrix Science, Boston, MA, USA) was used to identify peptide sequences present in a protein sequence database. The database search criteria were as follows: HMGB1 (CAG 33144.1, High Mobility Group Box 1), *Homo sapiens* (downloaded September 21, 2016) fixed modification, carbamidomethylated at cysteine residues, variable modification, oxidized at methionine residues, maximum allowed missed cleavage; 2, MS tolerance; 100 ppm, MS/MS tolerance; 0.1 Da. Only the peptides resulting from trypsin digestion were considered.

### Statistical analysis

The data were analyzed using one-way analysis of variance with Tukey’s honestly significant difference *post-hoc* test for multiple comparisons or a two-tailed Student’s *t*-test. Western blot image data were analyzed using image J software (National Institute of Mental Health, Bethesda, Maryland, USA). The average values are shown in the graphs. All values are presented as mean ± standard error of the mean (SEM) with *p* < 0.05 indicating statistical significance.

## Results

### USP13 Interacts with HMGB1

**Fig. 1:**
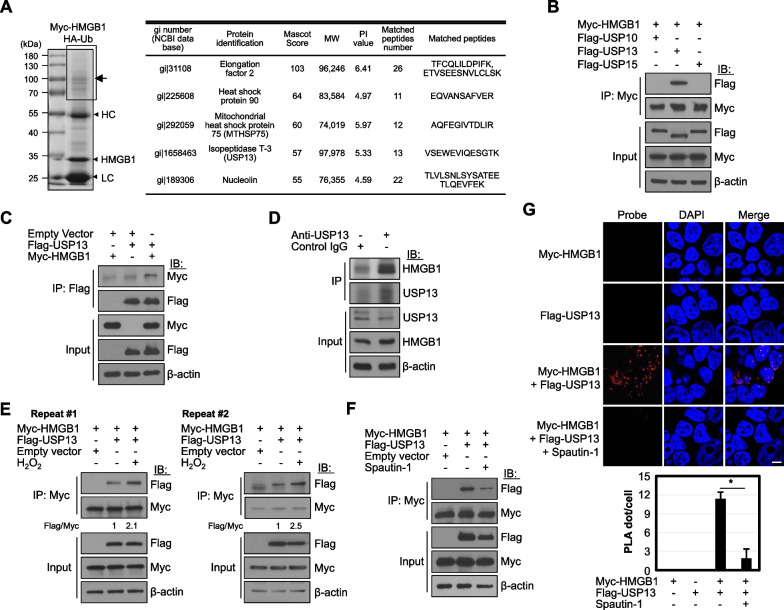
USP13 interacts with HMGB1. **A** HEK293T cells were co-transfected with both Myc-HMGB1 and HA-Ub plasmids for 48 h, and then treated with 10 μM MG132 for the last 18 h prior to harvest. WCLs were immunoprecipitated with anti-Myc affinity gel and separated by SDS-PAGE. Indicated box (arrow) was extracted for LC-MS/MS analysis. HC and LC: heavy and light chains. HMGB1 binding proteins were identified using LC-MS/MS for the analyses of peptides. **B** HEK293T cells were transfected with both Myc-HMGB1 and Flag-tagged USP10 or USP13 or USP15 plasmids and cultured for 48 h. WCLs were immunoprecipitated (IP) and immunoblotted (IB) with the indicated antibodies. **C** HEK293T cells were immunoprecipitated with anti-Flag antibody and immunoblotted with the indicated antibodies. **D** HEK293T cells were immunoprecipitated with anti-USP13 antibody, and the binding of endogenous HMGB1 was shown. **E**, **F** Both Myc-HMGB1 and Flag-USP13 were overexpressed in HEK293T cells, and cells were treated with 50 μM H_2_O_2_ **E** or 10 μM Spautin-1 **F** for the last 18 h before harvesting them to observe the binding. Data represents one of two similar independent experiments in **E**. **G** PLA was performed on HEK293T cells co-expressing Myc-HMGB1 and Flag-USP13 with 10 μM Spautin-1 treatment for last 18 h. For the PLA spot analysis, over 20 cells were counted. Scale bar: 10 μm. *p < 0.001 using Tukey’s honestly significant difference post-hoc test for multiple comparisons

HMGB1 has diverse roles depending on its various interactors (Bianchi 2009). LC-MS/MS analysis was performed to investigate the binding proteins related to ubiquitination. HEK293T cells were co-transfected with Myc-tagged HMGB1 (Myc-HMGB1) and HA-tagged Ub (HA-Ub) plasmids and treated with the proteasome inhibitor MG132. Myc-HMGB1 proteins were immunoprecipitated and separated by SDS-PAGE, and binding proteins were observed (Fig. 1A). Among several candidate proteins, heat shock protein 90 regulates the nucleocytoplasmic translocation of HMGB1 (Kim et al. 2021) and mitochondrial heat shock protein 75 (or glucose-regulated protein 75) is involved in HMGB1-induced asthmatic airway inflammation (Lv et al. 2018). In this study, we investigated the effect of isopeptidase T-3, also called USP13, on HMGB1, since HMGB1 ubiquitination has rarely been reported.

To confirm whether USP13 binds to HMGB1, both Myc-HMGB1 and Flag-tagged USP13 (Flag-USP13) or USP10 and USP15 plasmids were overexpressed in HEK293T cells for co-immunoprecipitation. USP10 was tested with a specific and potent autophagy inhibitor, Spautin-1, known to inhibit both USP13 and USP10 and regulate the deubiquitination of Beclin1 in Vps34 complexes, resulting in the promotion of the degradation of Vps34 complexes (Liu et al. 2011). As shown in Fig. 1B and 1C, HMGB1 binds to USP13 but not to either USP10 or USP15 after immunoprecipitation with Myc. HEK293T cells were immunoprecipitated with an anti-USP13 antibody for endogenous binding, and the binding of endogenous HMGB1 to USP13 is shown (Fig. 1D). In addition, their binding increased when ROS stress was induced (Fig. 1E). As expected, Spautin-1 treatment decreased the binding of HMGB1 to USP13, suggesting that inhibition of the deubiquitinase activity of USP13 affects its binding to HMGB1 (Fig. 1F). The interaction between HMGB1 and USP13 was also observed using a proximity ligation assay (PLA) in HEK293T cells overexpressing HMGB1 and USP13 (Fig. 1G).

### Binding domain study of HMGB1 and USP13

**Fig. 2:**
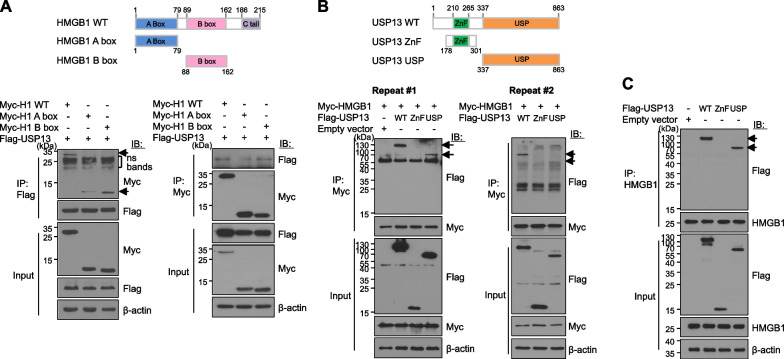
Binding domain study between HMGB1 and USP13. **A** HMGB1 wild type (WT), **A**, **B** boxes (upper panel). HEK293T cells were co-transfected with either Myc-HMGB1 A or B box and Flag-USP13 plasmid (lower panel). Immunoprecipitations (IPs) using anti-Flag and anti-Myc antibodies were done. ns: non-specific. WCLs were immunoprecipitated (IP) and immunoblotted (IB) with indicated antibodies. **B** USP13 WT, USP13 ZnF, and USP domains (upper panel). HEK293T cells were co-transfected with both Myc-HMGB1 and Flag-USP13 WT or USP13 ZnF or USP13 USP domain plasmids (lower panel). WCLs were immunoprecipitated (IP) and immunoblotted (IB) with indicated antibodies. **C** HEK293T cells were transfected with Flag-USP13 WT, ZnF, and USP domain plasmids alone, and immunoprecipitated with anti-HMGB1 Ab to confirm the binding of the domain of USP13

HMGB1 is composed of two homologous DNA-binding domains: A box and B box. When we transiently transfected HEK293T cells with both HMGB1 A (amino acid 1–79) or B (aa. 88–162) box and USP13 WT plasmids followed by either Flag or Myc IP, we found that the B box showed the weak binding to USP13 WT (Fig. 2A). By contrast, we generated two deletion mutant constructs, USP13-ZnF (aa. 178–301) and USP13-USP domains (aa. 336–863), and overexpressed these in HEK293T cells with the Myc-HMGB1 WT plasmid. IP analysis showed that the USP13-USP domain was weakly bound to HMGB1 WT (Fig. 2B). In addition, the endogenous HMGB1 binding to the USP13-USP domain was observed by HMGB1 IP (Fig. 2C).

### USP13 deubiquitinates HMGB1

**Fig. 3:**
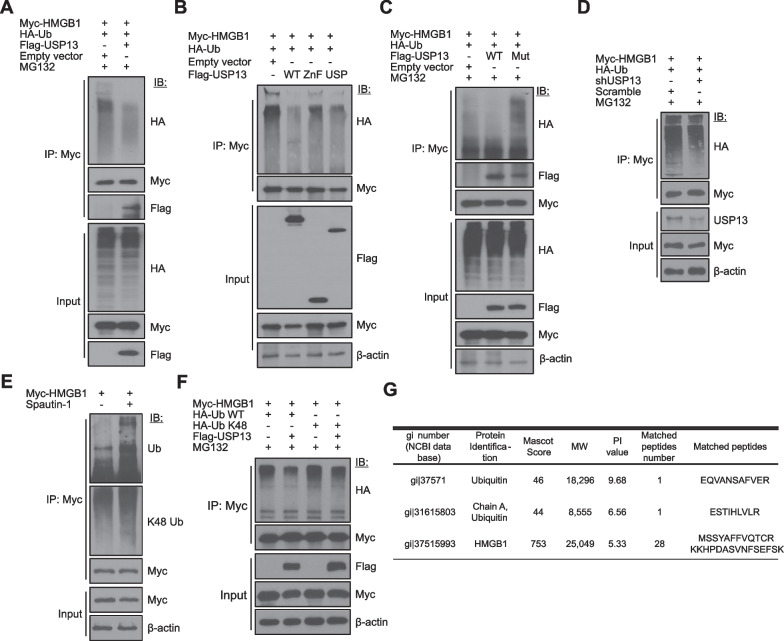
USP13 deubiquitinates HMGB1. **A**, **B** HEK293T cells were co-transfected with Myc-HMGB1, HA-Ub, and Flag-USP13 plasmids (**A**, N = 2) and with Myc-HMGB1, HA-Ub, Flag-USP13 wildtype (WT), ZnF or USP domain plasmids (**B**, N = 2) for 48 h. The cells were treated with 10 μM MG132 for the last 18 h before harvesting the cells. WCLs were immunoprecipitated (IP) and immunoblotted (IB) with the indicated antibodies. **C** HEK293T cells were co-transfected with indicated plasmids for 48 h and treated with MG132 for the last 18 h. Flag-USP13 WT and inactive USP13C345A/M664/739E (USP13 Mut) plasmids were used (N = 2). Data represents one of two similar independent experiments in **A**–**C**, respectively. **D** HEK293T cells were co-transfected with indicated plasmids for 48 h and treated with MG132 for the last 18 h. shUSP13 was used to knock down USP13 and scramble plasmid was used as a negative control. **E** The Myc-HMGB1 plasmid was overexpressed in HEK293T cells for 48 h, and the cells were treated with 10 μM Spautin-1 for the last 18 h; subsequently, IP and IB were performed. **F** HEK293T cells were co-transfected with the indicated plasmids for 48 h, and then treated with MG132 for the last 18 h, and IP and IB were performed to observe Ub-K48 modification. **G** HEK293T cells were co-transfected with Myc-HMGB1 and HA-Ub for 48 h and treated with MG132 for the last 18 h. WCL were immunoprecipitated with anti-Myc antibody and subjected to LC–MS/MS analyses

Subsequently, we tested whether USP13 could deubiquitinate HMGB1. HEK293T cells were co-transfected with both Myc-HMGB1 and Flag-USP13 plasmids along with the HA-Ub plasmid for 48 h and then treated with MG132, a proteasome inhibitor for the next 18 h before harvest. WCLs were immunoblotted with an anti-HA antibody after IP with an anti-Myc antibody. As shown in Fig. 3A, HMGB1 ubiquitination was decreased when USP13 was overexpressed. The USP13-USP domain, which binds to HMGB1 (Fig. 2B and C), mainly plays a role in HMGB1 deubiquitination (Fig. 3B). Next, we generated Flag-USP13 Mut, the USP13 enzymatically inactive triple mutant containing C345A, M664E, and M739E, to observe the restoration of ubiquitination. Each of the three mutated residue sites is a conserved motif in the ubiquitin-associated (UBA) domain of USP13 (Scortegagna et al. 2011; Sun et al. 2017; Zhang et al. 2011). HEK293T cells were co-transfected with Myc-tagged HMGB1, HA-Ub, Flag-USP13, or Flag-USP13 Mut and treated with MG132. The levels of HMGB1 K48-linked deubiquitination induced by Flag-USP13 overexpression were significantly recovered by an inactive mutant of Flag-USP13 Mut (Fig. 3C). HMGB1 ubiquitination was enhanced when USP13 levels were reduced by transfection with the shUSP13 plasmid (Fig. 3D). Furthermore, the inhibition of endogenous USP13 activity by Spautin-1 treatment confirmed HMGB1 ubiquitination (Fig. 3E). In fact, HMGB1 overexpression and Spautin-1 treatment also influenced the K48-linked ubiquitination of HMGB1 (Fig. 3E). We reconfirmed that K48-linked HMGB1 ubiquitination was reduced by USP13 overexpression when HEK293T cells were cotransfected with Myc-HMGB1 and HA-Ub K48 plasmids in the presence or absence of Flag-USP13 (Fig. 3F). This is supported by our previous data that *N*-glycosylation-defective mutants of HMGB1 (HMGB1^N37Q/N134Q^ and HMGB1^N37Q/N135Q^) undergo rapid degradation by K48-linked ubiquitination (Kim et al 2016). To investigate HMGB1 ubiquitination, HEK293T cells were co-transfected with Myc-HMGB1 and HA-Ub and then treated with MG132. LC-MS/MS was performed after immunoprecipitation with anti-Myc antibody. As shown in Fig. 3G, HMGB1 ubiquitination could be observed by LC-MS/MS analysis.

### Deubiquitination by USP13 increases HMGB1 stability

**Fig. 4:**
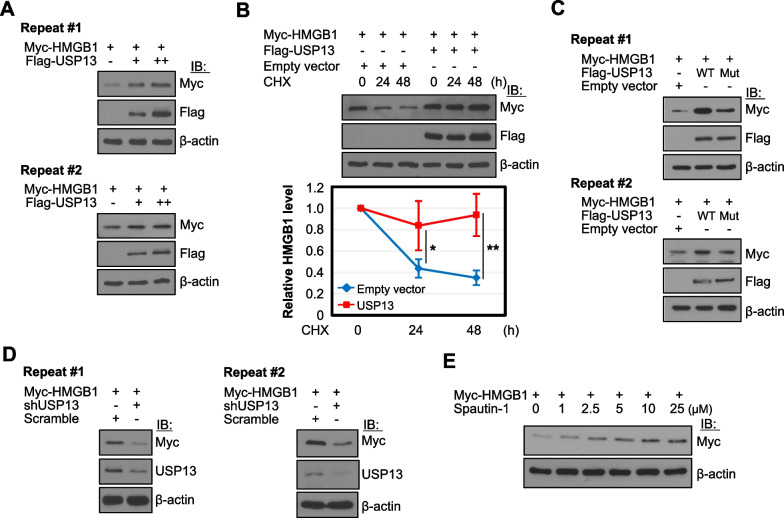
The levels of HMGB1 protein are increased by USP13. **A** HEK293T cells were co-transfected with both Myc-HMGB1 and various doses of Flag-USP13 plasmids and WCLs were then immunoblotted. **B** HEK293T cells were co-transfected with both Myc-HMGB1 and Flag-USP13 plasmids, and then treated with 100 μg/mL cycloheximide (CHX) for the indicated times and WCLs were immunoblotted. *p < 0.05, **p < 0.01 using two-tailed Student’s *t*-test. **C**, **D** HEK293T cells were co-transfected with Myc-HMGB1 and USP13C345A/M664/739E (USP13 Mut) **C** and shUSP13 for 48 h (**D**). WCLs were immunoblotted. **E** HEK293T cells were transfected with Myc-HMGB1 plasmid for 48 h, and then treated with Spautin-1 for the last 18 h indicated dose and WCLs were immunoblotted

To determine whether USP13 influences the stability of HMGB1, we overexpressed Myc-HMGB1 in HEK293T cells and then increased the number of Flag-USP13 plasmids. HMGB1 expression increased with high USP13 expression (Fig. 4A). HEK293T cells were treated with cycloheximide (CHX), a protein synthesis inhibitor, to observe HMGB1 stability after the transient overexpression of HMGB1 and USP13. The level of HMGB1 was sustained by the overexpression of USP13 compared to the empty plasmid, and the degradation rate of HMGB1 after USP13 overexpression was minimal compared to the empty vector control (Fig. 4B). Flag-USP13 Mut, however, failed to stabilize HMGB1 protein levels (Fig. 3C), indicating that USP13 regulates HMGB1 protein stability. Knockdown of USP13 expression by shUSP13 plasmid or inhibition of USP13 activity by Spautin-1 treatment decreased the stability of HMGB1 protein (Fig. 4D and E), demonstrating that USP13 regulates HMGB1 protein levels.

### USP13 drives the nucleocytoplasmic localization of HMGB1 and its secretion

**Fig. 5:**
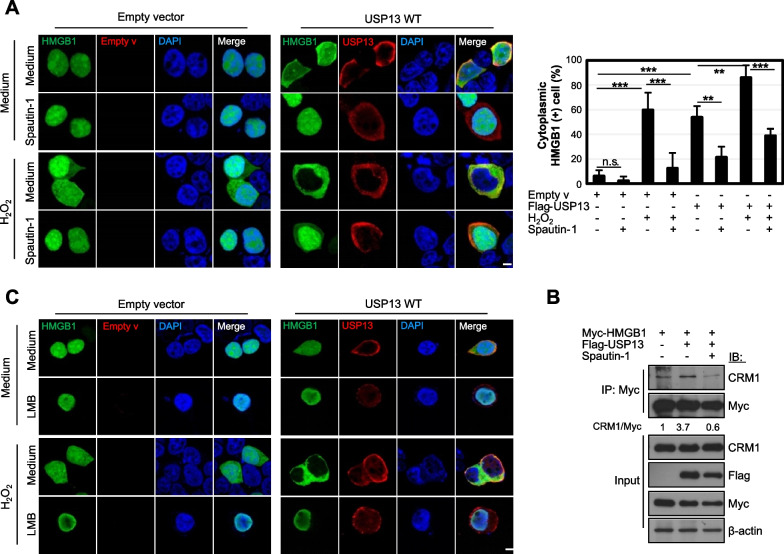
USP13 regulates subcellular localization of HMGB1. **A**, **C** HEK293T cells were co-transfected with both GFP-HMGB1 and Flag-USP13 or empty plasmids for 48 h. Cells were treated with 10 μM Spautin-1 for 2 h or 20 ng/mL leptomycin B (LMB) for 20 h and further treated with 50 μM H_2_O_2_ for 4 h. HMGB1 was observed after immunofluorescent staining. Over 100 cells were counted for cytoplasmic GFP-HMGB1 ( +) cells. Scale bar: 10 μm. *n.s.* no significant, N = 3. **p < 0.01 and ***p < 0.001 using Tukey’s honestly significant difference post-hoc test for multiple comparisons. **B** HEK293T cells were co-transfected with Myc-HMGB1 and Flag-USP13 plasmids for 48 h and treated with 10 μM Spautin-1 for the last 18 h and WCLs were immunoprecipitated (IP) with anti-Myc antibody and immunoblotted (IB) with anti-CRM1 antibody. Data represents one of two similar independent experiments in **B**

Some DUBs regulate the subcellular localization of target molecules; for example, p53 localization and stability are regulated by deubiquitinylation functioned by USP10, and the localization of PTEN are regulated by a HAUSP-PML network (Song et al. 2008; Yuan et al. 2010). We examined whether USP13 is related to the nucleocytoplasmic translocation of HMGB1. HEK293T cells were co-transfected with GFP-tagged HMGB1 (GFP-HMGB1), Flag-USP13, or empty plasmids. Interestingly, HMGB1 was translocated from the nucleus to the cytoplasm by USP13 overexpression, and this translocation was inhibited by Spautin-1 treatment (Fig. 5A). When cells were treated with 50 μM H_2_O_2_ after co-transfection with HMGB1 and USP13 plasmids, the nucleocytoplasmic translocation of HMGB1 was aggravated (Fig. 5A). These results showed that USP13 drives nuclear HMGB1 to migrate to the cytoplasm. To explore the mechanism of HMGB1 nucleocytoplasmic translocation, the binding of HMGB1 to CRM1, a nuclear export protein, was tested (Bonaldi et al. 2003). We observed that USP13 overexpression increased the interaction between HMGB1 and CRM1, and this binding was decreased by Spautin-1 treatment (Fig. 5B). In addition, LMB, a CRM1 inhibitor, inhibited the nucleocytoplasmic migration of HMGB1 following USP13 overexpression (Fig. 5C). These results suggest that USP13 increases HMGB1 nucleocytoplasmic translocation via a CRM1-dependent nuclear export pathway.

**Fig. 6:**
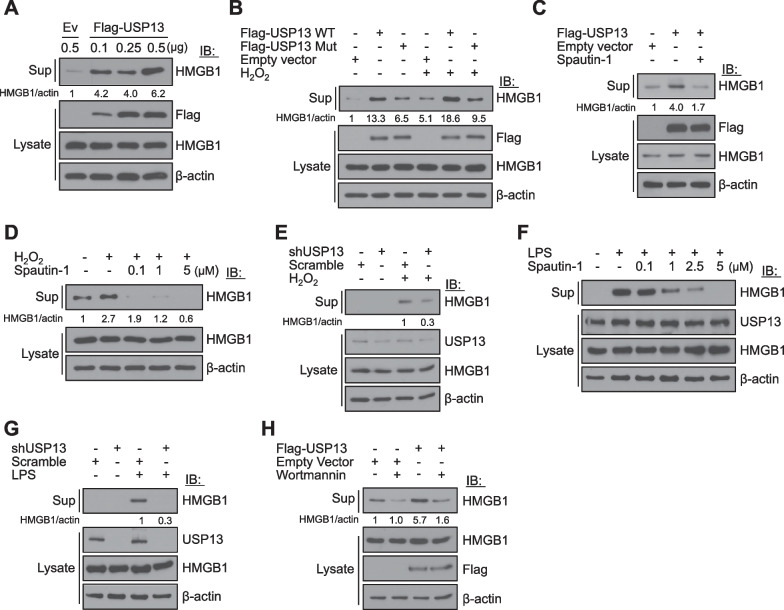
USP13 promotes HMGB1 secretion. **A** HEK293T cells were transfected with Flag-USP13 plasmid for 48 h. Culture supernatants were harvested and immunoblotted with anti-HMGB1 antibody. **B** HEK293T cells were overexpressed with USP13 wildtype (WT) or USP13C345A/M664/739E (Mut) plasmid for 48 h and treated with 50 μM H_2_O_2_ for the last 18 h. N = 3. **C** HEK293T cells were transfected with Flag-USP13 plasmid for 48 h and then treated with 10 μM Spautin-1 for the last 18 h. **D** HEK293T cells were pre-treated with 0.1, 1, 5 μM Spautin-1 for 1 h, and 50 μM H_2_O_2_ was treated for 18 h. **E** HEK293T cells were transfected with shRNA USP13 plasmid for 48 h and treated with 50 μM H_2_O_2_ for the last 18 h. **F** RAW 264.7 cells were pre-treated with 10 μM Spautin-1 for 1 h and then with 100 ng/mL LPS for 18 h. **G** Stably expressed HEK293/hTLR4-MD2-CD14 cells were transfected with shRNA USP13 plasmid for 48 h and treated with 100 ng/mL LPS for the last 18 h. **H** HEK293T cells were transfected with Flag-USP13 plasmid for 48 h and then treated with 80 nM Wortmannin for the last 24 h. All data represent one of two (**A**, **G**, and **H**) or three (**B**–**D**) similar independent experiments

**Fig. 7:**
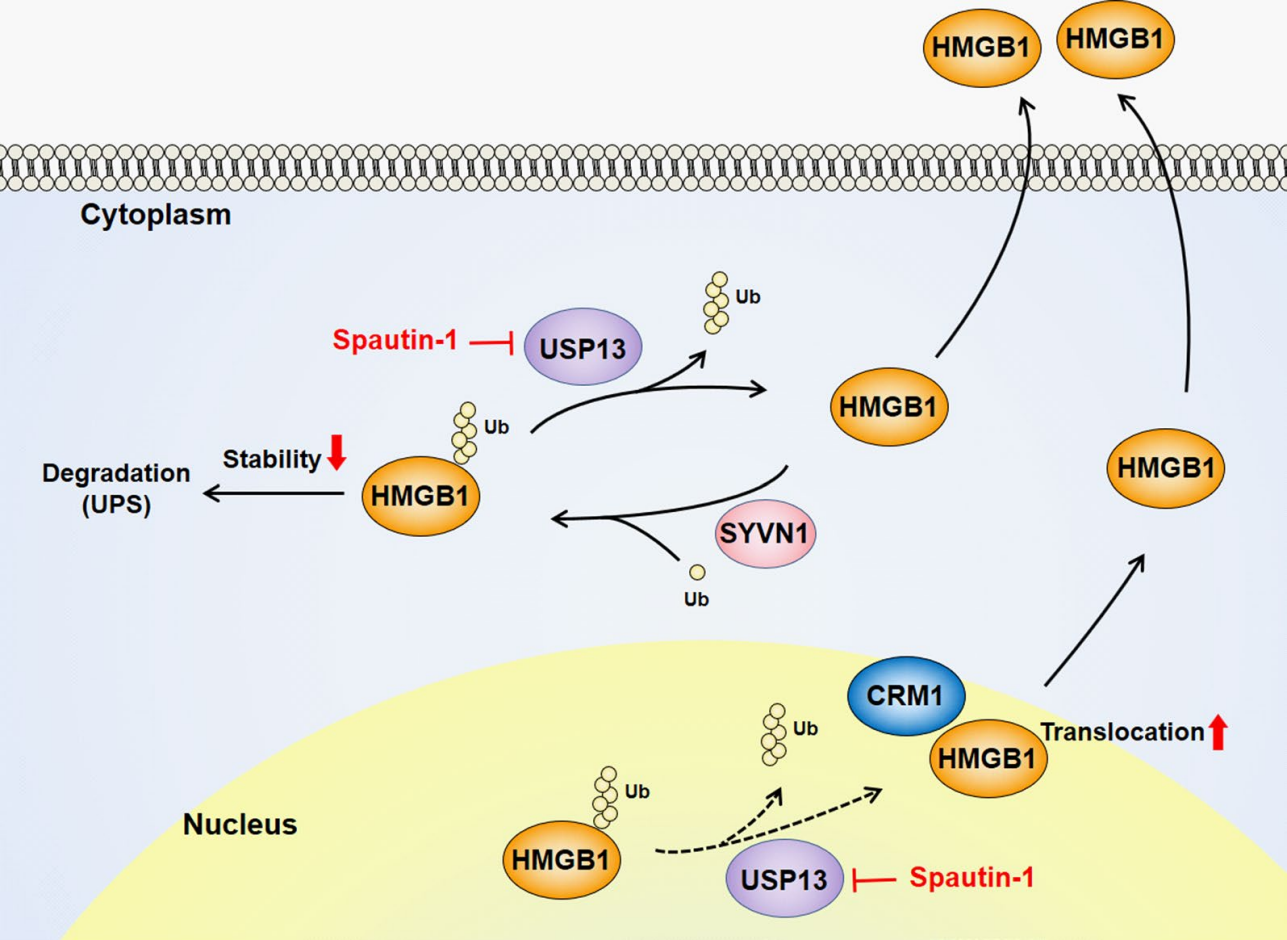
A model of the regulatory mechanism of HMGB1 by USP13. USP13 regulates HMGB1 stability and nucleocytoplasmic localization via CRM1-dependent pathway. Consequently, USP13 promotes HMGB1 secretion; this axis can be inhibited by Spautin-1. E3 ligase of HMGB1 which is used for ubiquitination is not known (X). Synoviolin (SYVN1) is reported as the E3 ligase of HMGB1 using bioinformatics analysis(W Yao et al. 2021). Dotted line is hypothetical pathway in nucleus. *UPS* ubiquitin–proteasome system

We further studied the effects of USP13 on HMGB1 secretion. HMGB1 secretion was promoted by USP13 overexpression in a dose-dependent manner (Fig. 6A). HMGB1 secretion was significantly reduced by USP13 Mut overexpression (Fig. 6B). Spautin-1 treatment inhibited USP13-mediated HMGB1 secretion (Fig. 6C). Interestingly, H_2_O_2_-stimulated HMGB1 secretion was inhibited by Spautin-1 treatment, in addition to inhibition by shUSP13 transfection (Fig. 6D and E). Furthermore, Spautin-1 inhibited LPS-stimulated HMGB1 secretion in RAW 264.7 cells, and shUSP13 transfection decreased HMGB1 secretion (Fig. 6F and G). Empirically, the secretory autophagy machinery is involved in HMGB1 secretion (Kim et al. 2021). Thus, wortmannin, a PI3-kinase inhibitor, decreased HMGB1 secretion in USP13-overexpressed cells (Fig. 6H). Collectively, our results suggest that USP13 deubiquitinates HMGB1 to maintain its stability and is a key molecule that regulates HMGB1 secretion (Fig. 7).

## Discussion

HMGB1 PTMs, including acetylation, phosphorylation, oxidation, and methylation, have been well-studied with respect to their secretion (Bonaldi et al. 2003; Hoppe et al. 2006; Ito et al. 2007; Kwak et al. 2019; Youn and Shin 2006). Previously, atypical *N*-glycosylation of HMGB1 was shown to be crucial for its nucleocytoplasmic translocation and secretion, and HMGB1 mutations at both Asn37 and Asn134 or 135 showed rapid degradation of HMGB1 by Ub-K48 linked ubiquitination (Kim et al. 2016).

In this study, we demonstrated, based on LC–MS/MS, immunoprecipitation, and PLA studies, that the deubiquitinase USP13 interacts with HMGB1. The HMGB1 B-box mainly binds to USP13 WT and the USP13-USP domain binds to HMGB1. HMGB1 is deubiquitinized by USP13 WT but not by inactive USP13 Mut or shUSP13, suggesting that HMGB1 deubiquitination is regulated by USP13. In addition, the level of HMGB1 protein was significantly increased by USP13 overexpression and decreased by shUSP13 or Spautin-1 treatment, showing that HMGB1 levels are regulated by the deubiquitinase USP13. Synoviolin (SYVN1), which is activated by histone demethylase KDM4D, was identified as an E3 ligase of HMGB1 using bioinformatics analysis for HMGB1 ubiquitination (Yao et al. 2021). SYVN1 induces HMGB1 polyubiquitination for degradation. Thus, HMGB1 ubiquitination and deubiquitination by SYVN1 and USP13, respectively, are important mechanisms for controlling the homeostasis of HMGB1. Recently, it was reported that USP12, another USP family member, is highly expressed in multiple myeloma cells and binds to HMGB1 to deubiquitinate and stabilize HMGB1, resulting in the promotion of pro-survival autophagy in myeloma (Li et al. 2022).

In addition, we found USP13 regulates HMGB1 translocation from the nucleus to the cytoplasm, resulting in its secretion. The translocation and secretion of HMGB1 were inhibited by shUSP13 and Spautin-1. Our data suggested that USP13 overexpression increased HMGB1 binding to CRM1 nuclear exportin for cytosolic translocation, resulting in its secretion. Considering that the nuclear ubiquitin–proteasome system plays a role in controlling gene expression and the quality control of nuclear proteins (von Mikecz 2006; von Mikecz et al. 2008), HMGB1 ubiquitination in the nucleus is probably involved in controlling gene regulation. LPS or H_2_O_2_ treatment showed almost no change of the level of USP13 expression in RAW264.7 cells in our study, so the mechanism of how proinflammatory stimulation influences HMGB1 secretion is related to USP13. Further study including the binding of USP13 to HMGB1 by proinflammatory stimulation is under investigation. In summary, we first identified the novel role of USP13 in deubiquitination-related HMGB1 secretion and homeostasis. To develop USP13 inhibitors is important to control HMGB1-related inflammation or diseases.

## Conclusion

USP13 can interact with Beclin1, deubiquitinate, and stabilize it (Liu et al. 2011). In addition, USP13 recruited by NEDD4-1 deubiquitinizes PIK3C3/VPS34 (phosphatidylinositol 3-kinase catalytic subunit type 3), which functions in autophagy initiation, resulting in the stabilization of PIK3C3 to promote autophagy (Xie et al. 2020). We previously demonstrated that secretory autophagy machinery and multivesicular body formation mediate HMGB1 secretion (Kim et al. 2021). Collectively, USP13 positively regulates HMGB1 secretion. In summary, we posit that USP13 is a major target for HMGB1 homeostasis and secretion.

## Data Availability

All data generated or analyzed during this study are included in this published article.

## Abbreviations

HMGB1: High mobility group box 1
DAMP: Damage-associated molecular pattern
LPS: Lipopolysaccharide
IL: Interleukin
TNF: Tumor necrosis factor
PTMs: Post-translational modifications
DUBs: Deubiquitinases
USPs: Ubiquitin-specific proteases
UCHs: Ubiquitin C-terminal hydrolases
OTUs: Ovarian tumor proteases
JAMMs: JAB1/MPN/Mov34 metalloenzymes
ZnF: Zinc finger
UIM: Ubiquitin-interacting motif
UBA: Ubiquitin-associated
USP13: Ubiquitin-specific protease 13
HEK: Human embryonic kidney
IB: Immunoblotting
IP: Immunoprecipitation
WT: Wild-type
PLA: Proximity Ligation Assay
LC-MS/MS: Liquid Chromatography with Tandem Mass Spectrometry
